# Targeting KSHV/HHV-8 Latency with COX-2 Selective Inhibitor Nimesulide: A Potential Chemotherapeutic Modality for Primary Effusion Lymphoma

**DOI:** 10.1371/journal.pone.0024379

**Published:** 2011-09-30

**Authors:** Arun George Paul, Neelam Sharma-Walia, Bala Chandran

**Affiliations:** H. M. Bligh Cancer Research Laboratories, Department of Microbiology and Immunology, Chicago Medical School, Rosalind Franklin University of Medicine and Science, North Chicago, Illinois, United States of America; Virginia Polytechnic Institute and State University, United States of America

## Abstract

The significance of inflammation in KSHV biology and tumorigenesis prompted us to examine the role of COX-2 in primary effusion lymphoma (PEL), an aggressive AIDS-linked KSHV-associated non-Hodgkin's lymphoma (NHL) using nimesulide, a well-known COX-2 specific NSAID. We demonstrate that (1) nimesulide is efficacious in inducing proliferation arrest in PEL (KSHV+/EBV-; BCBL-1 and BC-3, KSHV+/EBV+; JSC-1), EBV-infected (KSHV-/EBV+; Raji) and non-infected (KSHV-/EBV-; Akata, Loukes, Ramos, BJAB) high malignancy human Burkitt's lymphoma (BL) as well as KSHV-/EBV+ lymphoblastoid (LCL) cell lines; (2) nimesulide is selectively toxic to KSHV infected endothelial cells (TIVE-LTC) compared to TIVE and primary endothelial cells (HMVEC-d); (3) nimesulide reduced KSHV latent gene expression, disrupted p53-LANA-1 protein complexes, and activated the p53/p21 tumor-suppressor pathway; (4) COX-2 inhibition down-regulated cell survival kinases (p-Akt and p-GSK-3β), an angiogenic factor (VEGF-C), PEL defining genes (syndecan-1, aquaporin-3, and vitamin-D3 receptor) and cell cycle proteins such as cyclins E/A and cdc25C; (5) nimesulide induced sustained cell death and G1 arrest in BCBL-1 cells; (6) nimesulide substantially reduced the colony forming capacity of BCBL-1 cells. Overall, our studies provide a comprehensive molecular framework linking COX-2 with PEL pathogenesis and identify the chemotherapeutic potential of nimesulide in treating PEL.

## Introduction

KSHV is etiologically associated with PEL, an aggressive form of non-Hodgkin B-cell lymphoma (NHL) that accounts for 4% of all AIDS-associated NHLs (AIDS-NHL) with a poor prognosis and median survival of approximately six months [1], [2]. PEL, a transformed B cell of plasma cell lineage, is characterized by the expression of KSHV latency genes, unique clinical presentation and pathogenesis [1], [2]. Conventional chemotherapeutic regimens for similar aggressive NHLs provide no specific cure for PEL, although several lines of work are currently underway to develop anti-PEL therapies such as pro-apoptotic agents bortezomib and azidothymidine, anti-proliferative antibiotic rapamycin, p53 activator nutlin-3a, anti-viral compounds cidofovir and interferon-α, and KSHV latency gene blocking agents glycyrrhizic acid (GA) and small RNA transcripts [1], [3]–[12].

Non-steroidal anti-inflammatory drugs (NSAIDS) form one of the largest and most well studied groups of drugs with both anti-inflammatory and anti-cancer effects [13]–[21]. Although NSAIDs are traditionally used as anti-inflammatory and analgesic drugs, their anti-cancer potential is due to the direct correlation between elevated COX-2 and the pathogenesis of several cancers including colorectal, prostate, lung and breast cancers, as well as several hematological malignancies such as chronic lymphocytic leukemia, Hodgkin's and non-Hodgkin's lymphomas, and multiple myeloma [13]–[15], [22]–[24]. The oncogenic capacity of COX-2 is attributed to its ability to nurture diverse aspects of tumorigenesis such as proliferation, angiogenesis, blocking apoptosis, and metastasis, which have been well delineated at the molecular level in several models [13], [24]. However, the molecular mechanisms underlying COX-2 in AIDS related lymphomas such as PEL remains unresolved and the host mechanisms utilized by KSHV in PEL pathogenesis is an active area of investigation. Since the current treatment regimens for PEL are not effective and have severe life threatening side effects, we rationalize that a valuable strategy with improved outcomes in immunocompromised patients would be the one that selectively targets viral oncogenes, induces apoptosis of infected cells and eradicates the latent virus load. Coupled with the immense anti-cancer properties of NSAIDs and COX-2's known role in oncogenesis, we predicted that understanding the role of COX-2 in PEL, if any, might pave the way for identifying a unique arena of drug targets for treating PEL that can act as anti-viral and anti-cancer drugs [13], [24]–[29].

In the present study, we demonstrate the relevance of COX-2 in PEL latency and the chemotherapeutic potential of the COX-2 inhibitor nimesulide in treating PEL. Nimesulide (4-nitro-2-phenoxymethanesulphonanilide) is an orally active COX-2 selective inhibitor. Nimesulide produces potent analgesic, anti-inflammatory and antipyretic activities *in vivo*. It has been reported to produce fewer gastrointestinal side effects than standard NSAIDs such as diclofenac, naproxen and ketoprofen [19], [30]–[31]. To examine the effect of nimesulide treatment, we employed the largest possible set of patient-derived NHL cell lines published to date. These cell lines included PEL (BCBL-1, BC-3, JSC-1), EBV-infected (Raji) and non-infected (Akata, Loukes, Ramos, BJAB) human Burkitt's lymphoma (BL), EBV harboring lymphoblastoid (LCL), KSHV infected (TIVE-LTC), TIVE, and primary endothelial (HMVEC-d) cells. Our studies uncovered the potent responsiveness of KSHV infected PEL cell lines towards COX-2 inhibition via nimesulide treatment compared to lines with EBV co-infection or highly aggressive KSHV-/EBV- BL cell lines. Nimesulide treatment blocked KSHV latency genes LANA-1 and vFLIP, LANA-1/p53 interaction, induced apoptosis, G1 cell cycle arrest and down-regulated survival kinases as well as the angiogenic factor VEGF-C. Nimesulide reduced the transforming properties of BCBL-1 cells as observed by a decreased number of colonies formed on soft agar. The drug also down-regulated the expression of genes uniquely overexpressed in PEL cells such as vitamin-D3 receptor (VDR), aquaporin-3 and syndecan-1 (CD138). Effective inhibition of PEL pathogenesis and survival related events strongly suggest that COX-2 blockade by the well-characterized clinically approved COX-2 inhibitor nimesulide has immense potential as a chemotherapeutic agent against PEL and warrants further investigation in a PEL-SCID xenograft murine model.

## Materials and Methods

### Cell cultures

PEL (KSHV+/EBV-; BCBL-1 and BC-3, KSHV+/EBV+; JSC-1), EBV-infected (KSHV-/EBV+; Raji) and non-infected (KSHV-/EBV-; Akata, Loukes, Ramos, BJAB) human Burkitt lymphoma (BL) and KSHV-/EBV+ lymphoblastoid (LCL - primary B cells transformed by B95-8 EBV) cell lines were cultured in RPMI 1640 (Gibco BRL, Grand Island, New York) medium with 10% heat-inactivated fetal bovine serum (FBS; HyClone, Logan, Utah), 2 mM L-glutamine (Gibco BRL), and penicillin/streptomycin (Gibco BRL). BC-3, JSC-1 and BJAB cells were purchased from ATCC, Manassas, VA. Akata, LCL, Loukes, and Raji cell lines were a kind gift from Dr. Lindsey Hutt-Fletcher (Louisiana State University Health Sciences Center, Shreveport). BCBL-1 cell line was a kind gift from Dr. McGrath (The University of California, San Francisco). KSHV-BJAB (KSHV+/EBV-) was cultured in the same media with 0.2 mg/ml hygromycin B purchased from Sigma, St. Louis, Mo [32]. TIVE and TIVE-LTC (long term infected telomerase immortalized umbilical vein endothelial cells) were a gift from Dr. Rolf Renne (University of Florida). TIVE, TIVE-LTC and HMVEC-d (human dermal microvascular endothelial cells) cells were cultured in EBM-2 (Lonza Walkersville) with growth factors [28].

### Reagents

Nimesulide, indomethacin, and diclofenac were from Sigma. NS-398 was from Calbiochem, La Jolla, CA. DAPI and rabbit-cleaved-caspase 3 antibody were from Invitrogen, Carlsbad, CA. Propidium iodide (PI) was from BD Biosciences, San Jose, CA.

### Antibodies

COX-1 and COX-2 antibodies were from Cayman Chemicals, Ann Arbor, MI. Rabbit LANA-1 antibody has been described before [28]. p-GSK-3β, GSK-3β, p-cdc2, cdc2, and cdc25C antibodies were from Cell Signaling Technology, Inc., Beverly, MA. Cyclin D1, cdk6, cyclin E, cyclin A, p21 (cip1/waf-1), p53, aquaporin-3, VDR, CD138/syndecan-1, and β-actin antibodies were from Santa Cruz Biotechnology, Inc., Santa Cruz, CA. Alexa-488 or 594-coupled anti-mouse antibodies were from Molecular Probes, Eugene, OR. Conjugates of anti-mouse/rabbit-alkaline phosphatase and anti-mouse/rabbit-horseradish peroxidase were from Kirkegaard and Perry Laboratories, Inc., Gaithersburg, MD.

### Measurement of PGE2, Western blotting, and immunofluorescence

Secreted amounts of PGE2 were measured using a PGE2 ELISA kit as per the manufacturer's guidelines (R & D systems, Minneapolis, MN). Total cell lysates prepared from cells after their respective treatments were used for Western blotting and quantified as described before [29]. Immunofluorescence was conducted as described before and the images presented are high-resolution deconvoluted images [28] with no nearest neighbors.

### Immunoprecipitation assay

5×10^7^ BCBL-1 cells were used to isolate nuclear lysates at indicated time points and treatments using the Nuclear complex Co-IP kit supplied by Active Motif (Carlsbad, CA). Nuclear lysates were then used for immunoprecipitation (low stringency) to examine LANA-1-p53 complexes as described by Chen et al., 2010 [33].

### Fluorescent activated cell sorting (FACS)

Samples for FACS analysis were prepared as per manufacturer's guidelines (BD Biosciences). The data were collected using a flow cytometry analysis using a LSRII (Becton Dickinson, Bedford, MA) and analyzed with FlowJo software at the RFUMS flow cytometry core facility.

### Cell cycle analysis by FACS

BCBL-1 and BJAB cells were fixed in 70% ethanol overnight and DNA was stained with propidium iodide at a final concentration of 50 µg/ml with RNaseA (100 U/ml) prior to analysis by FACS and ModFit Lt V3 software (Verity Software House).

### Real-time reverse transcription PCR (RT-PCR)

LANA-1, COX-2, and COX-1 transcripts were detected by real-time RT-PCR as described before [28]. The primer sequences for various genes are as follows: a) vFLIP (forward, 5′-AGGTTAACGTTTCCCCTGTTAGC-3′; reverse, 5′-AGCAGGTCGCGCAAGAG-3′), ORF50 (forward, 5′-CGCAATGCGTTACGTTGTTG-3′; reverse, 5′-GCCCGGACTGTTGAATCG-3′), AQP-3 (forward, 5′-GGAATAGTTTTTGGGCTGTA-3′; reverse, 5′-GGCTGTGCCTATGAACTGGT-3′), autotaxin (forward, 5′-ACAACGAGGAGAGCTGCAAT; reverse, 5′-AGAAGTCCAGGCTGGTGAGA-3′), CD138/syndecan-1 (forward, 5′-GGAGCAGGACTTCACCTTTG-3′; reverse, 5′-CTCCCAGCACCTCTTTCCT-3′), VDR (forward, 5′-CTTCAGGCGAAGCATGAAGC-3′; reverse, 5′-CCTTCATCATGCCGATGTCC-3′), VEGF-A (forward, 5′-CTTGCCTTGCTGCTCACC-3′; reverse, 5′-CACACAGGATGGCTTGAAG-3′), VEGF-C (forward, 5′-AGATGCCTGGCTCAGGAAGA-3′; reverse, 5′-TGTCATGGAATCCATCTGTTGA-3′), IL-10 (forward, 5′-GCCGTGGAGCAGGTGAAG-3′; reverse, 5′-GAAGATGTCAAACTCACTCATGGCT-3′), actin (forward, 5′-TCACCCACACTGTGCCATCTACGA-3′; reverse, 5′-CAGCGGAACCGCTCATTGCCAATGG-3′), GAPDH (forward, 5′-GAAGGTGAAGGTCGGAGTC-3′; reverse, 5′-GAAGATGGTGATGGGATTTC-3′). Normalization was done with respect to 18 s mRNA levels [26].

### Cell proliferation, cytotoxicity, apoptosis, and viability assays

1–5×10^5^ of the indicated cells were seeded with respective drugs at the indicated concentrations and the number of viable cells were examined by measuring their metabolically active mitochondria (an index of cell proliferation) based on a colorimetric assay (ATCC, Manassas, VA) as per the manufacturer's instructions at the indicated time points. Number of live BCBL-1 cells was determined by manually counting after respective treatments using traditional trypan blue staining (evaluation of cell membrane integrity) in quadruplicate counts. Supernatants of TIVE, HMVEC-d and TIVE-LTC cells treated with 50 µM and 100 µM nimesulide were collected at the indicated time points to assess cellular toxicity using a cytotoxic assay kit (Promega, Madison, WI) as previously described [28]. Levels of cleaved-caspase-3 levels were measured after indicated treatments using Apoalert caspase colorimetric assay (Clontech, Mountain View, CA) and by FACS analysis.

### Colony formation assay

5×10^5^ BCBL-1 cells were used to evaluate the effect of 100 µM nimesulide on the colony forming capacity of BCBL-1 cells and to measure their proliferative capacity within the colonies as per the manufacturer's guidelines using an MTT based CytoSelect cell transformation assay (Cell Biolabs, Inc., San Diego, CA).

### Measurement of GSK-3β and Akt

5×10^5^ of BCBL-1 cells treated with 100 µM nimesulide were used to measure the levels of p-GSK3β, t-GSK3β, p-Akt, and t-Akt as per the manufacturer's instructions at the indicated time points by Fast Activated Cell-based ELISA (FACE) kits for AKT and GSK-3β.

### Statistical analysis

For multiple comparisons, one-way ANOVA with Tukey's posthoc comparisons were used to assess the statistical significance of differences between means (p<0.05). For all other data analysis, a student's t-test was employed.

## Results

### KSHV+ NHL cell lines demonstrate greater proliferative vulnerability compared to KSHV- cell lines

**Figure 1:**
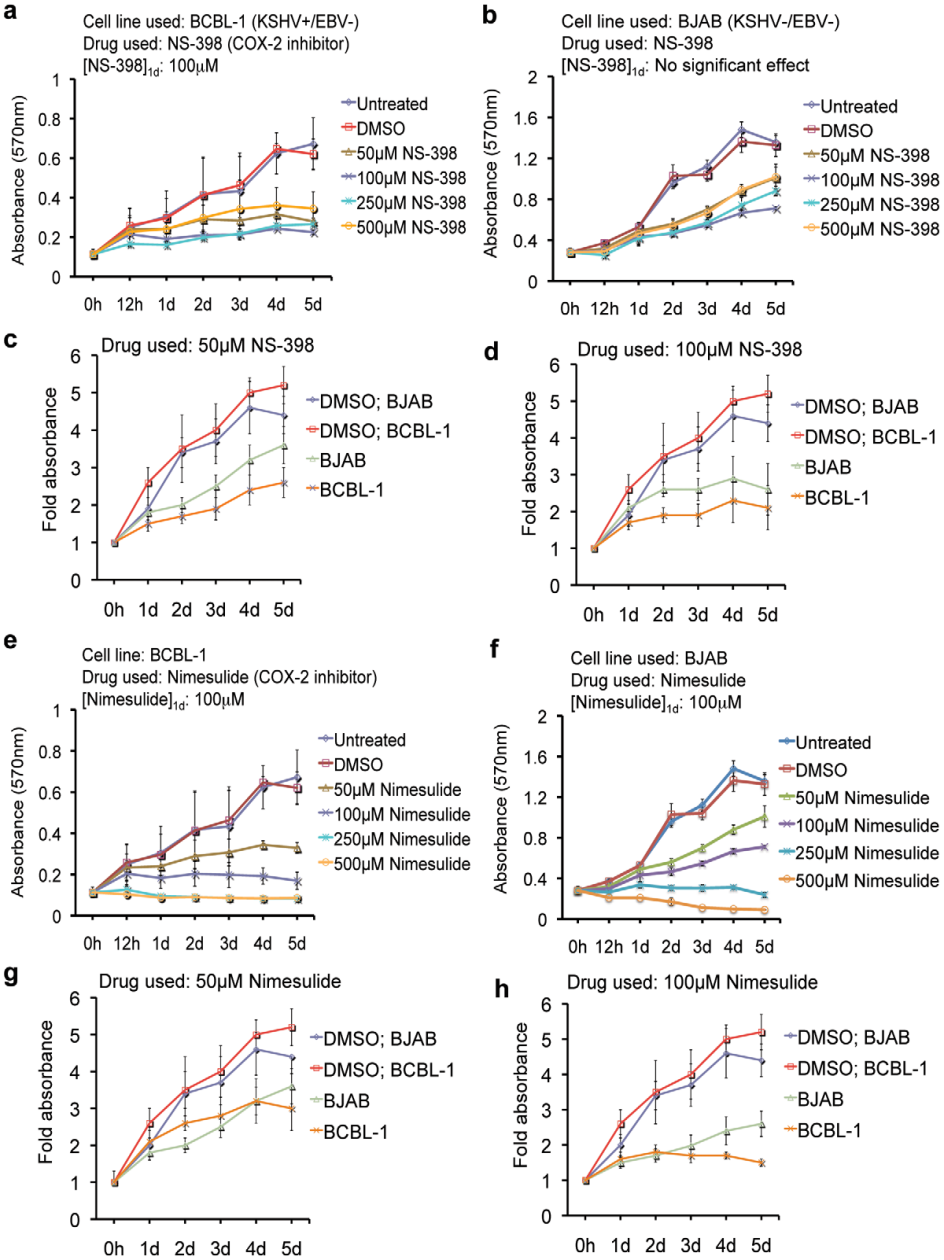
Sensitivity of KSHV + PEL cells to COX-2 specific inhibitors. (**a–h**) 5×10^5^ BCBL-1 or BJAB cells were seeded with the indicated concentrations of COX-2 inhibitors NS-398 (**a–d**), nimesulide (**e–h**), DMSO (**a–h**) or left untreated (**a–h**). Cell proliferation was measured by MTT assay at 0 h, 12 h, day 1 (1d), day 2 (2d), day 3 (3d), day 4 (4d), and day 5 (5d). The cells were neither replenished with fresh media nor supplemented with the drugs. (**c, d, g, h**) Plots comparing the proliferation rate of BCBL-1 and BJAB cells treated with 50 µM and 100 µM of NS-398 and nimesulide 0 h, 1d, 2d, 3d, 4d, and 5d post-treatment. The fold absorbance was calculated with respect to the untreated (UN) control before treatment. (**a–h**) One-way ANOVA with Tukey's posthoc comparison analysis (p<0.05) was used to determine whether the drug treatment induced a statistically significant difference in the proliferative indexes at 1d, 2d, 3d, 4d and 5d compared to untreated cells of the respective cell lines. Indicated on the graphs are the concentrations of each drug that had a significant effect at 1d ([drug]_1d_). Each reaction was done in quadruplicate, and each point represents the average ± S.D. from four independent experiments.

**Figure 2:**
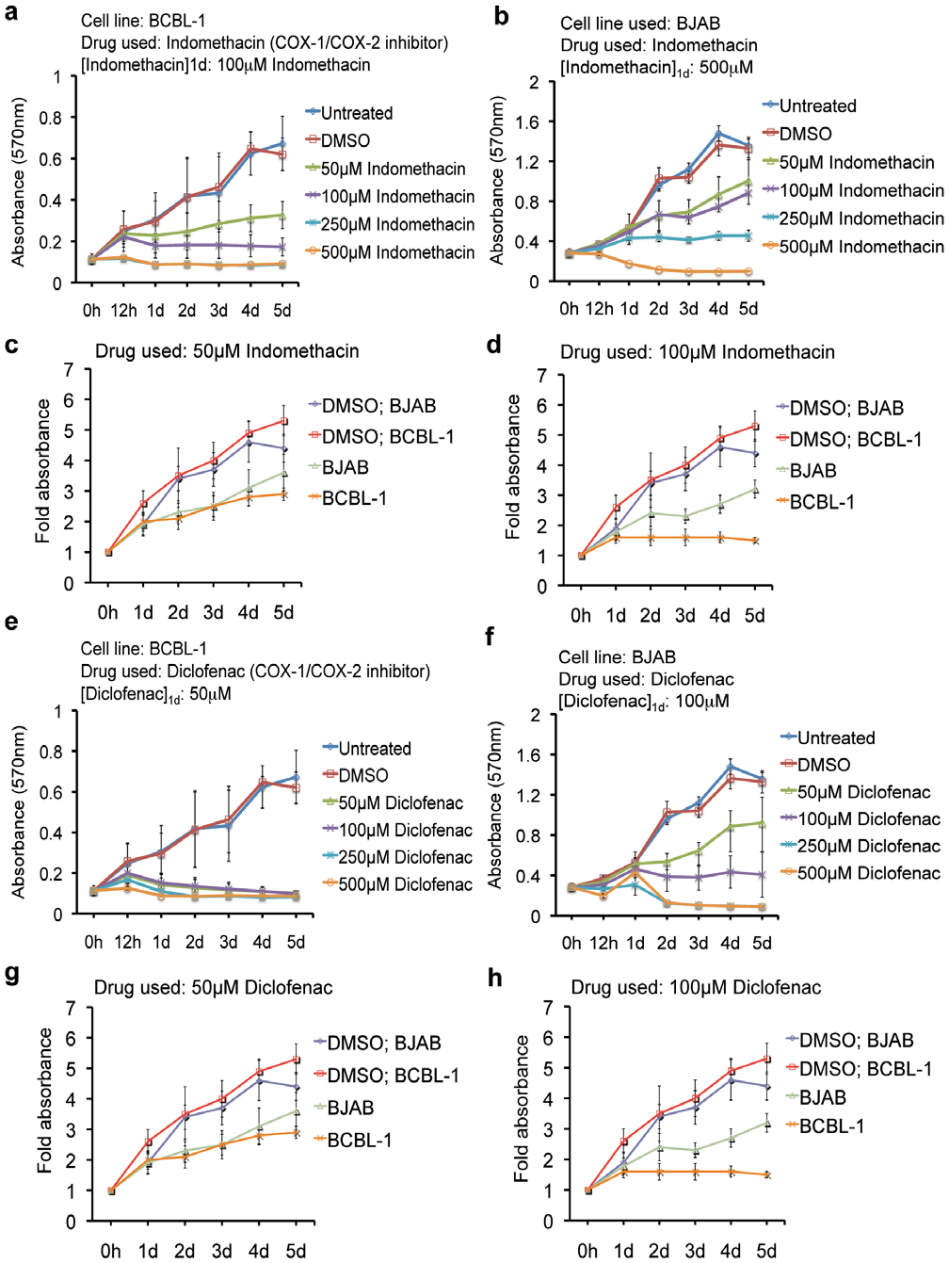
Sensitivity of KSHV + PEL cells to COX-1/COX-2 inhibitors. (**a–h**) 5×10^5^ BCBL-1 or BJAB cells were seeded with the indicated concentrations of COX-1/COX-2 inhibitors indomethacin (**a–d**) and diclofenac (**e–h**), DMSO (**a–h**) or left untreated (**a–h**). Cell proliferation was measured by MTT assay at 0 h, 1d, 2d, 3d, 4d, and 5d. The cells were neither replenished with fresh media nor supplemented with the drugs. (**c, d, g, h**) Plots comparing the proliferation rate of BCBL-1 and BJAB cells treated with 50 µM and 100 µM of indomethacin and diclofenac at 1d, 2d, 3d, 4d, and 5d. The fold absorbance was calculated with respect to the untreated (UN) control before treatment. (**a–h**) One-way ANOVA with Tukey's posthoc comparison analysis (p<0.05) was used to determine whether the drug treatment induced a statistically significant difference in the proliferative indexes at 1d, 2d, 3d, 4d, and 5d compared to untreated cells of the respective cell lines. Indicated on the graphs are the concentrations of each drug that had a significant effect at 1d ([drug]_1d_). Each reaction was done in quadruplicate, and each point represents the average ± S.D. from four independent experiments.

We first screened the effects of different NSAIDs on NHL cell lines BCBL-1 (KSHV+/EBV-) and BJAB (KSHV-/EBV-) using COX-2 inhibitors NS-398 and nimesulide as well as COX-1/COX-2 inhibitors indomethacin and diclofenac by MTT assay. Statistical analysis showed that at 2d, 3d, 4d, and 5d post-treatment, 50 µM was the lowest concentration required to induce a significant reduction in the proliferation rate of both BCBL-1 and BJAB cells for all of the drugs tested (Fig. 1 and Fig. 2). However, at 1d post-treatment 100 µM NS-398 had a significant reduction in BCBL-1 cell growth (Fig. 1a), and in contrast, had no effect on BJAB proliferation even at 500 µM (Fig. 1b). Similarly, nimesulide (Fig. 1e) and diclofenac (Fig. 2e) at 50 µM and indomethacin (Fig. 2a) at 100 µM had a significant reduction in BCBL-1 cell growth but not in BJAB cells 1d post-treatment. However, BJAB cell proliferation was significantly reduced 1d post-treatment at higher concentrations of nimesulide (250 µM; Fig. 1f), indomethacin (500 µM; Fig. 2b), and diclofenac (500 µM; Fig. 2f). We also compared the proliferative rate of both cell types on the same plot with 50 µM and 100 µM treatments of various NSAIDs (Fig. 1c, 1d, 1g, 1h, 2c, 2d, 2g, and 2h). A visible difference was not observed at 1d and 2d with both 50 µM and 100 µM treatments indicating that BJAB and BCBL-1 are both sensitive to 100 µM of NS-398, nimesulide, indomethacin, and diclofenac at 3d, 4d, and 5d (Fig. 1c, 1d, 1g, 1h, 2c, 2d, 2g, and 2h).

**Figure 3:**
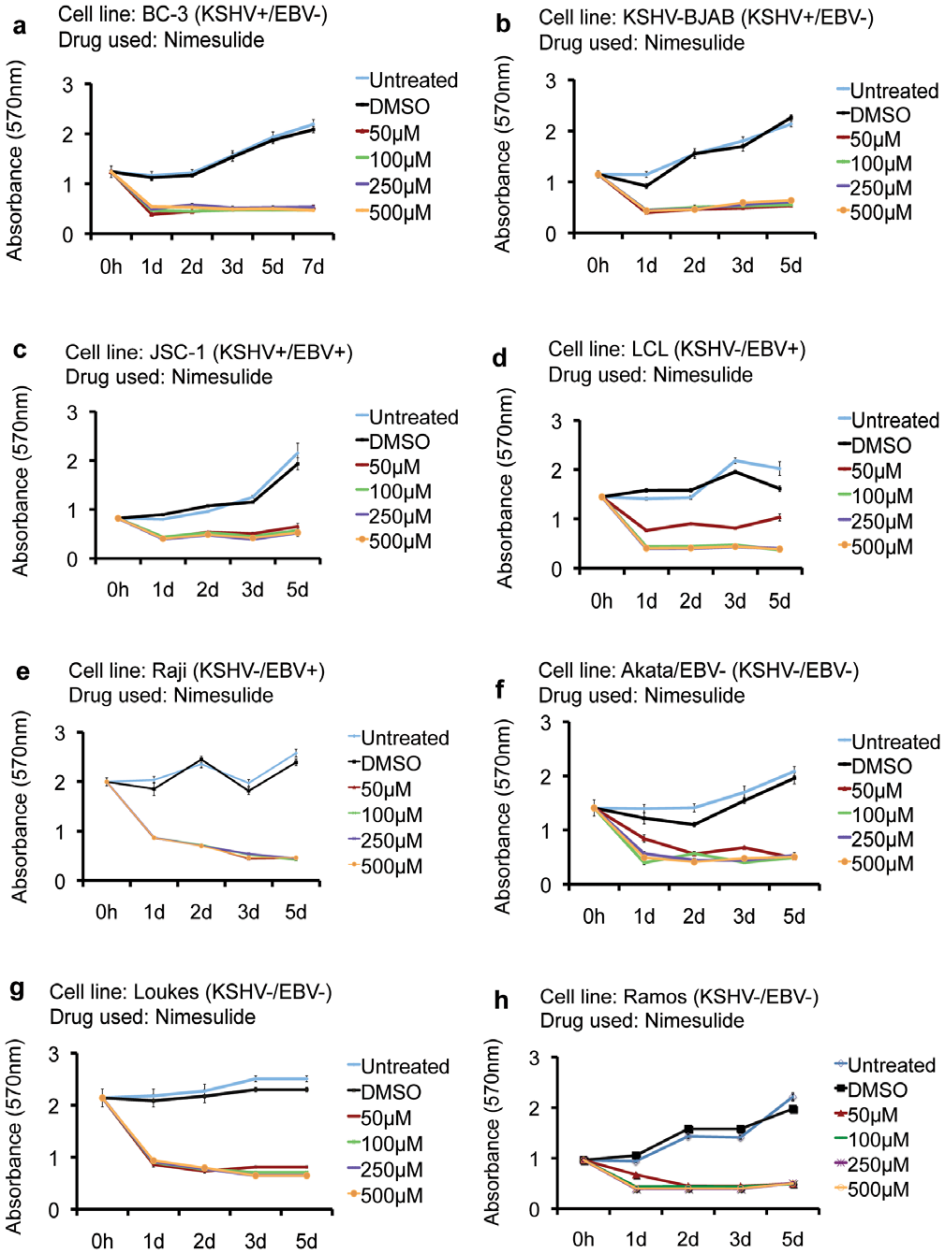
Effect of nimesulide on various NHL cell lines. (**a–h**) 5×10^5^ BC-3, KSHV-BJAB, JSC-1, LCL, Raji, Akata/EBV-, Loukes and Ramos cells were seeded with the indicated concentrations of nimesulide (**a–h**), DMSO (**a–h**), left untreated (**a–h**). The cell proliferation index was measured by MTT assay at 0 h, day 1 (1d), day 2 (2d), day (3d), and day 5 (5d). The cells were neither replenished with fresh media nor supplemented with the drugs. (**a–h**) One-way ANOVA with Tukey's posthoc comparison analysis (p<0.05) was used to determine whether the drug treatment induced a statistically significant difference in the proliferative indexes at 1d, 2d, 3d, and 5d compared to untreated cells of the respective cell lines. Each reaction was done in quadruplicate, and each point represents the average ± S.D. from three independent experiments.

Among the different NSAIDs tested, the drug with known specificity for COX-2 and that had the most potent effect on BCBL-1 was nimesulide. Therefore, we next examined whether the anti-proliferative effects of nimesulide on BCBL-1 (Fig. 1e) is replicable on other PEL cell lines. Our data demonstrate that 50 µM-500 µM nimesulide was able to induce significant proliferative arrest on KSHV+/EBV- PEL cells BC-3 (Fig. 3a) and KSHV-BJAB (Fig. 3b). Our data also demonstrates that similar to BCBL-1, BC-3, and KSHV-BJAB, 50 µM, 100 µM, 250 µM, and 500 µM of nimesulide was able to induce significant proliferation arrest in the KSHV+/EBV+ cell line JSC-1, KSHV-/EBV+ cell lines Raji and LCL, and KSHV-/EBV- cell lines Ramos, Loukes, and Akata/EBV- as well (Fig. 3c–h).

To examine whether there is a statistically significant difference on the potency of the anti-proliferative effects of nimesulide on the different cell lines tested, we conducted Tukey's posthoc comparison analysis between the proliferative indexes of the different cell lines used 1 day post-treatment with 50 µM nimesulide (Table 1). The analysis was done on cell lines (BCBL-1, BC-3, KSHV-BJAB, JSC-1, LCL, Ramos, BJAB, and Akata/EBV-) with no statistically significant difference on the basal proliferation index before drug treatment at 0 h (data not shown). Our data indicates that compared to KSHV-/EBV- cell lines (Ramos, Akata/EBV-), the proliferative index of KSHV+/EBV- cell lines (BCBL-1, BC-3, KSHV-BJAB), and the KSHV+/EBV+ cell line JSC-1 but not the KSHV-/EBV+ cell line LCL was significantly more decreased by nimesulide treatment at 1d with 50 µM nimesulide (Table 1) but not at 2d, 3d, and 5d (data not shown). Similarly, the proliferation index of BCBL-1, BC-3, KSHV-BJAB, and JSC-1 was significantly more decreased compared to KSHV-/EBV+ LCL cells (Table 1). Table 1 also indicates that the proliferation index of similarly infected cell lines BCBL-1, BC-3, KSHV-BJAB and BJAB, Ramos, and Akata/EBV- were not significantly different. Our analysis did not show any significant difference between the proliferation indexes at 1d, 2d, 3d, and 5d post-treatment with 100 µM, 250 µM, and 500 µM nimesulide treatments between any of the cell lines (data not shown).

**Table 1:** *Tukey posthoc* analysis of the differences in the means of the proliferative capacity of different cell lines at 1 day post-treatment with 50 µM Nimesulide.

	Cell lines compared (Cell line 1 vs cell line 2)	p-Value	Statistical significance (+) Significant (p<0.01)(−) Not significant (p≥0.01)
**KSHV+/EBV-** **vs** **KSHV-/EBV-**	BC-3 vs BJAB	2.12E-08	+
	BC-3 vs Akata/EBV-	1.78E-08	+
	BC-3 vs Ramos	5.92E-05	+
	BCBL-1 vs BJAB	3.03E-08	+
	BCBL-1 vs Akata/EBV-	2.25E-08	+
	BCBL-1 vs Ramos	6.40E-04	+
	KSHV-BJAB vs BJAB	3.03E-08	+
	KSHV-BJAB vs Akata/EBV-	2.25E-08	+
	KSHV-BJAB vs Ramos	6.40E-04	+
**KSHV+/EBV+** **vs** **KSHV-/EBV-**	JSC-1 vs BJAB	2.74E-08	+
	JSC-1 vs Akata/EBV-	2.14E-08	+
	JSC-1 vs Ramos	4.18E-04	+
**KSHV+/EBV-** **vs** **KSHV-/EBV+**	BCBL-1 vs LCL	6.49E-07	+
	BC-3 vs LCL	6.57E-08	+
	KSHVBJAB vs LCL	9.31E-08	+
**KSHV+/EBV+ vs KSHV-/EBV+**	JSC-1 vs LCL	4.01E-07	+
**KSHV+/EBV+** **vs** **KSHV+/EBV-**	JSC-1 vs BC-3	0.99998	−
	JSC-1 vs BCBL-1	1	−
	JSC-1 vs KSHVBJAB	1	−
**KSHV-/EBV+** **vs** **KSHV-/EBV**-	LCL vs BJAB	0.99209	−
	LCL vs Akata/EBV-	0.91085	−
	LCL vs Ramos	0.83948	−
**KSHV+/EBV-** **vs** **KSHV+/EBV**-	BCBL-1 vs BC-3	0.99987	−
	KSHVBJAB vs BC-3	1	−
	KSHVBJAB vs BCBL-1	0.99998	−
**KSHV-/EBV-** **vs** **KSHV-/EBV**-	Akata/EBV- vs BJAB	0.99999	−
	Ramos vs BJAB	0.22771	−
	Ramos vs Akata/EBV-	0.08016	−

Overall, our data strongly suggests that KSHV infected NHLs were more vulnerable to nimesulide mediated COX-2 blockade. To examine the COX-2 mediated mechanisms utilized by KSHV in PEL pathogenesis, we used BCBL-1 as a representative cell line for the remainder of the study and nimesulide as the NSAID of choice. Nimesulide was introduced in 1985 and since then, it is a well-studied drug that is already prescribed to approximately 500 million people in 50 different countries [14], [19], [30]. The reported *in vitro* IC50 of nimesulide for most cancer cell lines has been reported to be more than 150 µM-175 µM [31], [34]. Tukey's posthoc comparison analysis of the mean proliferative indexes at day 1 between the different concentrations of nimesulide demonstrated that 100 µM had a more significant decrease in the proliferative capacity of BCBL-1 cells compared to 50 µM (data not shown). Therefore, based on Tukey's posthoc comparison analysis, we chose 100 µM as the drug concentration for treatment throughout our study. We did not supplement the drug in our treatment regimen.

### Nimesulide down-regulates proliferation of TIVE-LTC but not TIVE and HMVEC-d cells

To delineate whether the anti-proliferative effects of nimesulide on various KSHV+ NHL cell lines is due to the presence of KSHV infection coupled with the known anti-growth effects of nimesulide or due to a non-specific cytotoxic effect of the drug, we next compared the effect of nimesulide on the proliferation of TIVE-LTC (KSHV+), TIVE (KSHV-), and primary endothelial (HMVEC-d) cells. Nimesulide significantly down-regulated the proliferation capacity of TIVE-LTC cells (Figure S1b) with no significant effect on the proliferation of TIVE (Figure S1a) and HMVEC-d (Figure S1c).

### Nimesulide inhibits the colony forming capacity of BCBL-1 cells

**Figure 4:**
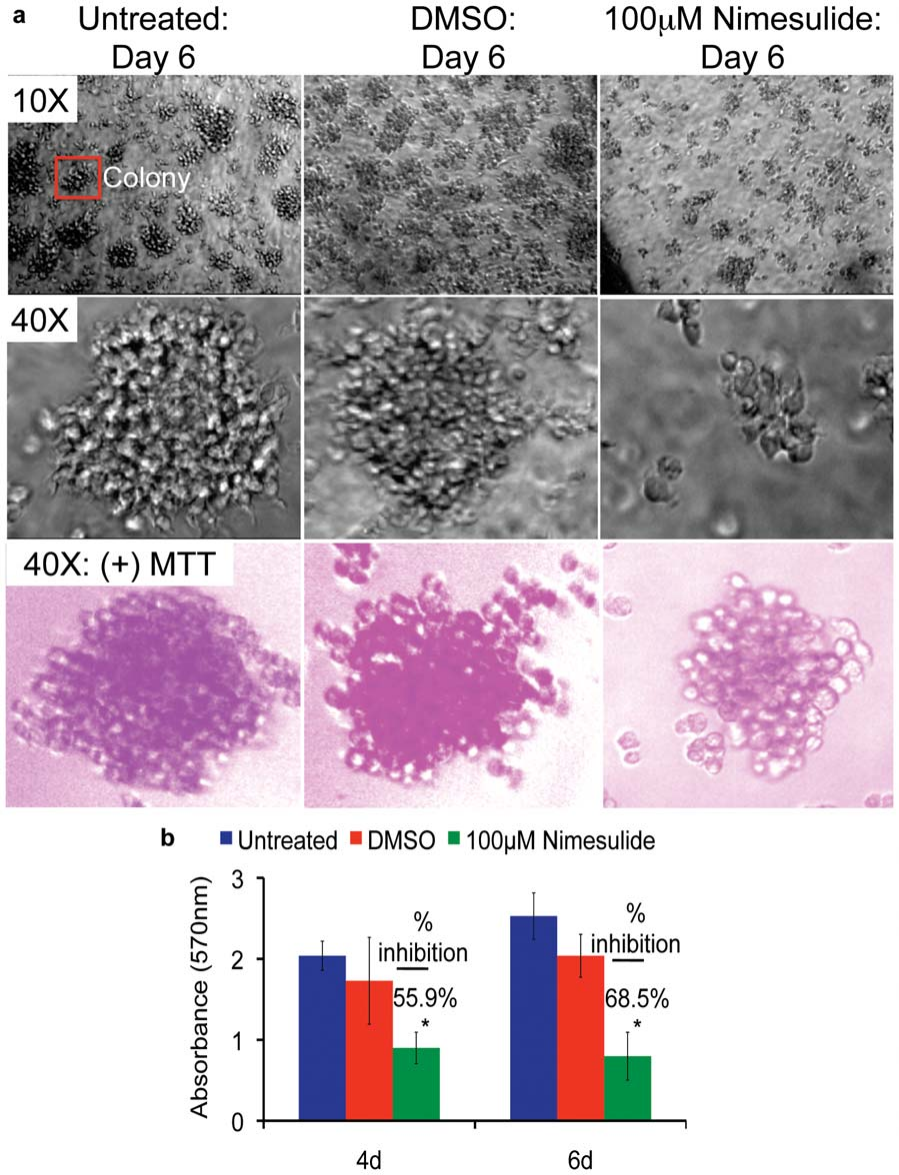
Effect of nimesulide on the transforming properties of BCBL-1 cells. (**a–b**) 5×10^5^ BCBL-1 cells seeded with 100 µM nimesulide and colony formation was evaluated by a cell transformation assay. The cells were neither replenished with fresh media nor supplemented with the drugs. 6d (day 6) post-treatment with nimesulide, 10× and 40× bright field images were taken with (a; bottom panel) and without (a; top and middle panel) MTT and the proliferative capacity of cells within the colonies were measured using MTT at 4d (day 4) and 6d post treatment (b). Each experiment was done in triplicate, and each point represents the average ± S.D. from three independent experiments. (*) p<0.05, (**) p<0.01.

KSHV has been shown to have transforming and oncogenic potential [35]. BCBL-1 is a fully transformed NHL cell line and its oncogenic capacity to form colonies *in vitro* on soft agar has been studied. In the next part of our study, we examined the effect of nimesulide on the colony forming capacity of BCBL-1 cells. Compared to untreated and DMSO treated cells, nimesulide treatment decreased the number of the characterstic adherent colony forming units 4d (data not shown) and 6d post-treatment on soft agar (Fig. 4a; top and middle panel). Quantification of the proliferation rate of BCBL-1 cells within colonies by MTT strongly indicates that nimesulide significantly decreased the colony forming capacity of BCBL-1 cells at 4d and 6d post-treatment (Fig. 4b). Qualitatively, this is further demonstrated by the decreased MTT uptake by nimesulide treated cells (Fig. 4a; bottom panel).

### Nimesulide down-regulates KSHV latency genes

**Figure 5:**
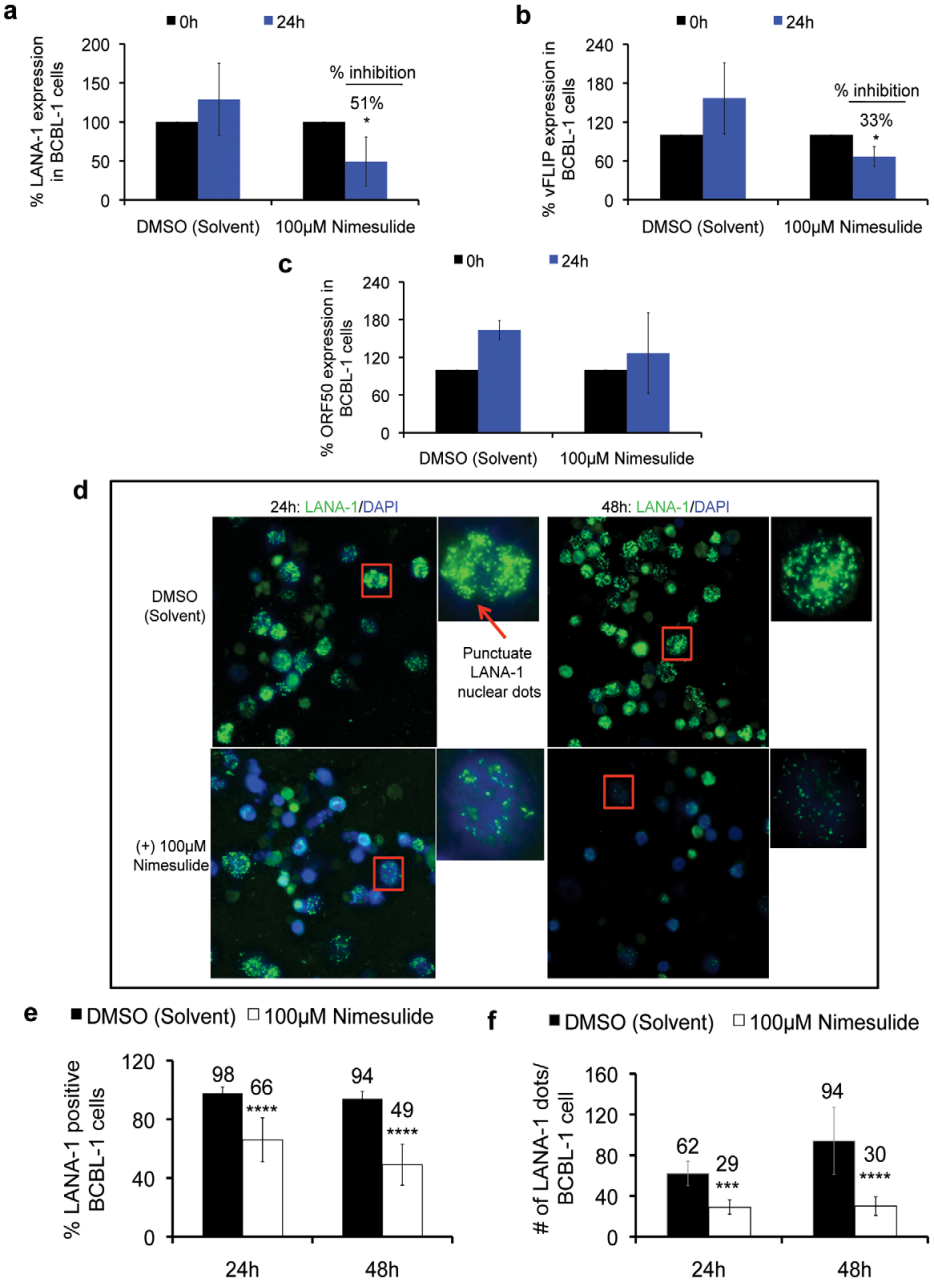
Effect of nimesulide on KSHV gene expression. (**a–e**) 5×10^5^ BCBL-1 cells were seeded with 100 µM nimesulide, DMSO or left untreated (UN) without replenishment with fresh media. RNA was isolated at 24 h post-treatment with the drug to examine the gene expression levels of LANA-1 (**a**), v-FLIP (**b**), and ORF50 (**c**) by real-time RT-PCR. The cells were neither replenished with fresh media nor supplemented with the drugs. The percent inhibition and statistics (t-test) were calculated with respect to untreated for each treatment (*) p<0.05. (**d–e**) In parallel experiments, LANA-1 protein was examined by immunofluorescence using anti-LANA-1 antibody (**d**) and used to calculate the percentage of cells positive for LANA-1 (**e**) at 24 h and 48 h post-treatment from 500 randomly selected cells. (**f**) Levels of LANA-1 protein per cell were measured by examining the number of LANA-1 dots/BCBL-1 cell by manually counting the number of ‘punctate nuclear LANA-1 dots’ in 20 BCBL-1 cells positive for LANA-1 from each treatment. (**e–f**) The indicated means are the averages calculated from cells selected from different fields by two observers. The corresponding statistics (t-test) were calculated with respect to DMSO treatment for each time point. (**a–c, e, f**) Each experiment was done in tripicate, and each point represents the average ± S.D. from three independent experiments (*) p<0.05, (****) p<5*10^−10^.

We next examined the effect of blocking COX-2 on viral latent and lytic gene expression. At 24 h post-treatment, nimesulide significantly down-regulated latent LANA-1 (Fig. 5a) and vFLIP (Fig. 5b) gene expression by 51% and 33%, respectively, with no effect on ORF50 (Fig. 5c), actin and GAPDH expression (data not shown). Next we determined the effect of 24 h and 48 h nimesulide treatment (100 µM) on LANA-1 protein levels by immunofluorescence in BCBL-1 cells. The DMSO treated BCBL-1 cells showed nuclear staining for LANA-1 with its characteristic ‘punctuate nuclear dot pattern’ in 98% and 94% of the cells at 24 h and 48 h post-treatment, respectively (Fig. 5d–e). In contrast, nimesulide treatment significantly decreased the LANA-1 positive cells to 66% and 49% at 24 h and 48 h post-treatment, respectively (Fig. 5d–e). When we quantified LANA-1 protein expression on a per BCBL-1 cell basis by counting the number of ‘LANA-1 dots’ on cells positive for LANA-1, nimesulide treatment significantly decreased the LANA-1 dots/cell to 29 and 30 dots/cell from 62 and 94 dots/cell in DMSO treated cells at 24 h and 48 h post-treatment, respectively (Fig. 5f). In summary, these results demonstrated that the COX-2 inhibitor nimesulide down-regulated the expression of KSHV latency genes (LANA-1 and vFLIP) and LANA-1 protein without affecting the expression of the master lytic cycle regulator ORF50.

### Nimesulide altered cell cycle regulatory proteins in BCBL-1 cells

**Figure 6 pone-0024379-g006:**
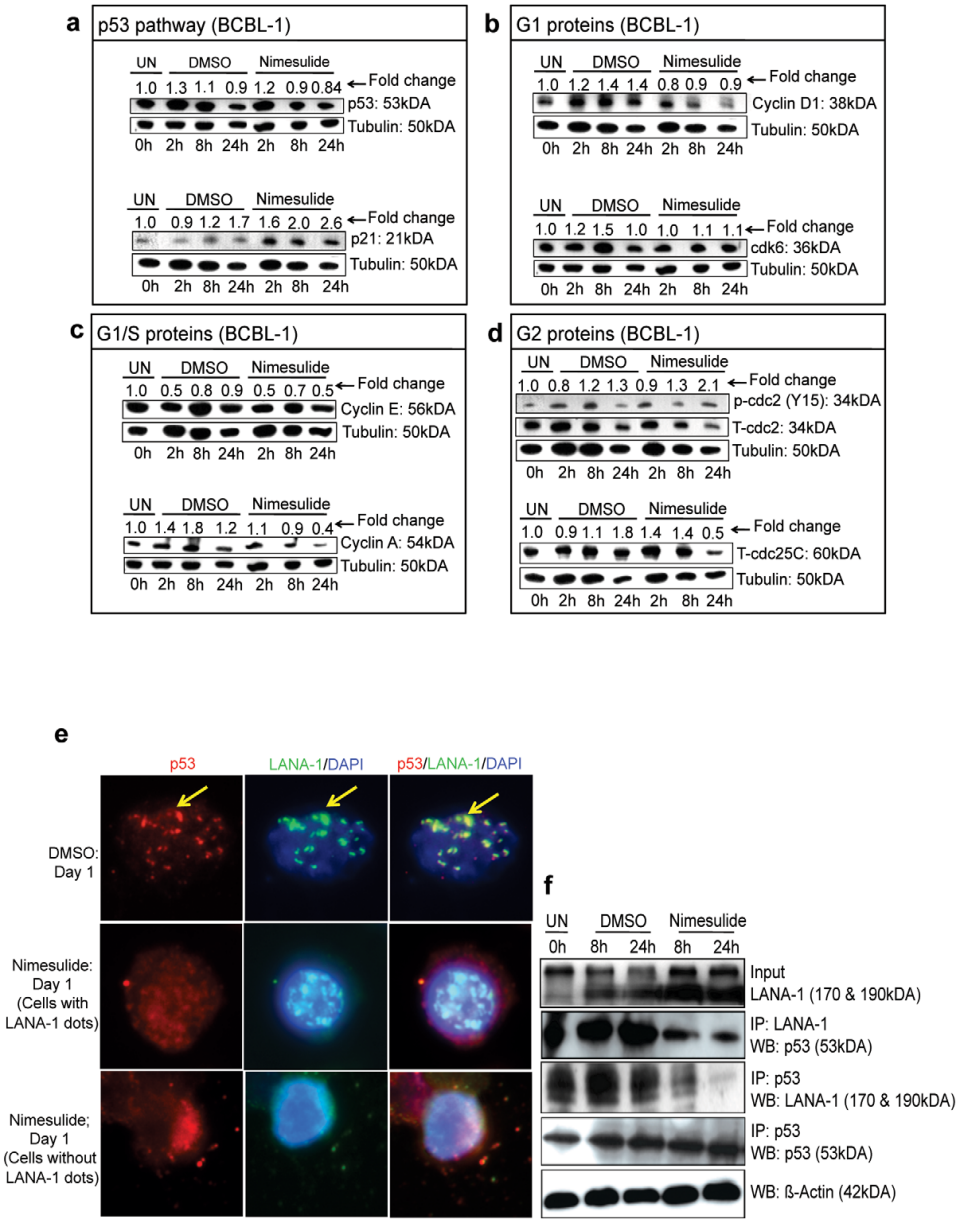
Effect of nimesulide on cell cycle regulatory proteins and LANA-1/p53 interaction. (**a–d**) 5×10^5^ BCBL-1 cells were seeded with 100 µM nimesulide or DMSO. Total cell lysates were collected at 2 h, 8 h, and 24 h post-treatment and immunoblotted for p53 (**a**), p21 (**a**), cyclin D1 (**b**), cdk6 (**b**), cyclin E (**c**), cyclin A (**c**), p-cdc2 (**d**), and T-cdc25C (**d**) and normalized with respect to the tubulin loading control and T-cdc2 for the p-cdc2 blot. The cells were neither replenished with fresh media nor supplemented with the drugs. The fold change was calculated with respect to untreated (UN) cells at 0 h. (**a–d**) The data is representative of duplicate experiments. (**e**) 5×10^5^ BCBL-1 cells were seeded with 100 µM nimesulide or DMSO and collected to examine the interaction between p53 and LANA-1 by immunofluorescence 24 h post-treatment. (**f**) Nuclear lysates isolated from BCBL-1 cells seeded with 100 µM nimesulide, DMSO or left untreated (UN) were immunoprecipitated (IP) with either LANA-1 or p53 and Western blotted (WB) with p53 and LANA-1 or p53, respectively. Level of LANA-1 in pre-IP input was assessed by Western blotting with LANA-1 antibody. β-actin was used as the loading control. (**e–f**) The cells were neither replenished with fresh media nor supplemented with the drugs.

BCBL-1 cells were seeded with either DMSO or 100 µM nimesulide and total lysates were collected at 2 h, 8 h, and 24 h to measure the levels of p53 and p21, G1 proteins cyclin D1 and cdk6, G1/S proteins cyclin E and cyclin A, and G2 proteins p-cdc2 and cdc25C. Compared to DMSO treatment, nimesulide had no effect on p53 but induced p21 at 2 h, 8 h, and 24 h by about 1.6, 2.0, and 2.6-fold, respectively (Fig. 6a). Next, we examined the G1 proteins cyclin D1 and cdk6 as well as the G1/S proteins cyclin E and cyclin A, which are required for progression through G1 and for the transition to S phase, respectively. We did not observe any changes in cyclin D1 and cdk6 between DMSO and nimesulide treatments (Fig. 6b). However, at 24 h post-treatment compared to DMSO, nimesulide down-regulated cyclin E and cyclin A by about 50% and 80%, respectively, (Fig. 6c). When we analyzed the effect of nimesulide on the G2 protein p-cdc2, which is de-phosphorylated by the phosphatase cdc25C, for progression of the cell cycle from G2 to the mitotic phase, at 24 h post-treatment, cdc25C was down-regulated by about 50% with a corresponding increase in p-cdc2 by 2.1-fold (Fig. 6d).

### Nimesulide releases p53 from LANA-1 mediated sequestration in BCBL-1 cells

Our results showing the down-regulation of LANA-1 protein (Fig. 5d–f), the activation of the major p53 response gene p21 (Fig. 6a), and the absence of any change in total p53 protein levels (Fig. 6a) led us to investigate whether nimesulide is functionally activating p53 by blocking the LANA-1-p53 interaction. BCBL-1 cells seeded with 100 µM nimesulide were collected at 24 h to examine the LANA-1-p53 interaction by immunofluorescence. DMSO treated cells demonstrated the characteristic ‘punctate nuclear dots’ phenotype for p53 colocalizing with LANA-1 (Fig. 6e, top panels). However, nimesulide treatment completely abolished this effect with the dispersal of p53 staining within the nucleus and in the cytoplasm (Fig. 6e, bottom panels). We also observed dispersed p53 staining in cells with the characteristic ‘punctate LANA-1 dots’ without any colocalization with LANA-1 indicating that nimesulide abolishes the LANA-1-p53 interaction (Fig. 6e, middle panels).

We further examined the stability of the LANA-1/p53 complex with nimesulide treatment with a series of immunoprecipitation (IP) assays. Reduction in LANA-1/p53 complex at 8 h and 24 h post-treatment with 100 µM nimesulide is strongly demonstrated by the co-IP and reverse co-IP experiments with LANA-1 and p53 antibodies with no significant changes in the DMSO treatments compared to untreated (Fig. 6f).

### Nimesulide down-regulates cell survival kinases in BCBL-1 cells

**Figure 7:**
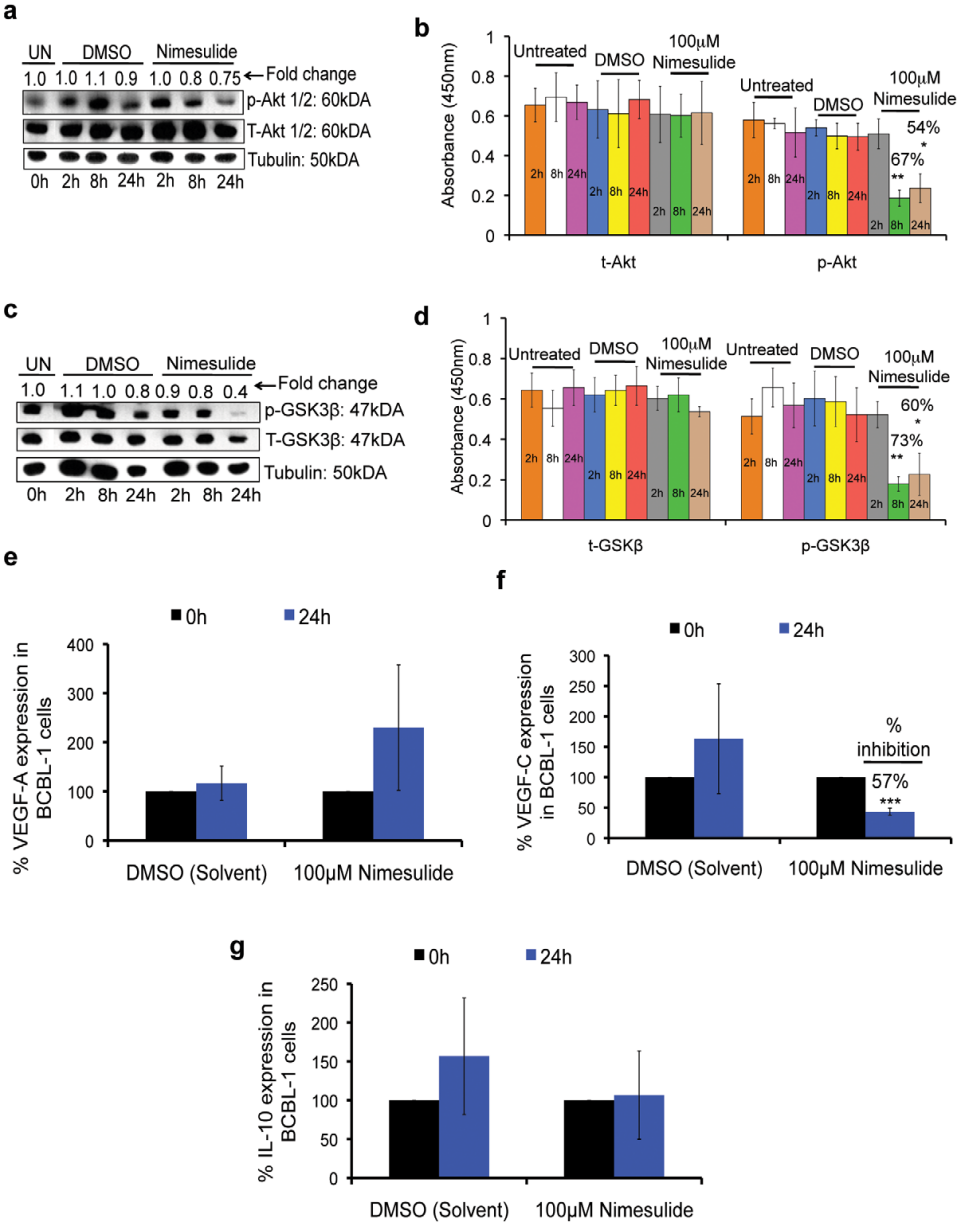
Effect of nimesulide on p-Akt, p-GSK-3β, IL-10, VEGF-A, and VEGF-C. (**a and c**) 5×10^5^ BCBL-1 cells were seeded with 100 µM nimesulide or DMSO and total lysates were collected at 2 h, 8 h, and 24 h post-treatment and immunoblotted for p-Akt 1/2 (**a**) and p-GSK-3β (**c**) and normalized with respect to t-Akt 1/2 and t-GSK-3β, respectively. Tubulin was used as the loading control for each blot. The cells were neither replenished with fresh media nor supplemented with the drugs. The fold change was calculated with respect to untreated (UN) cells at 0 h. The data is representative of duplicate experiments. (**b and d**) 5×10^5^ BCBL-1 cells were seeded with 100 µM nimesulide, DMSO or left untreated and samples were collected at 2 h, 8 h, and 24 h post-treatment to measure t-Akt (**b**), p-Akt (**b**), p-GSK-3β (**d**), and t-GSK-3β (**d**) by FACE assay. The cells were neither replenished with fresh media nor supplemented with the drugs. (**e–f**) 5×10^5^ BCBL-1 cells were seeded with 100 µM nimesulide or DMSO or left untreated (UN) without replenishment with fresh media. RNA was isolated at 24 h post-treatment with the drug to examine the gene expression levels of VEGF-A (**e**), VEGF-C (**f**), and IL-10 (**g**) by real-time RT-PCR. The cells were neither replenished with fresh media nor supplemented with the drugs. The fold change and corresponding statistics (t-test) were calculated with respect to untreated (UN) cells for each treatment. (***) p<0.0005. (**b, d, e, f, g**) Each experiment was done in tripicate, and each point represents the average ± S.D. from three independent experiments.

We next examined the effect of blocking COX-2 on major cell survival proteins such as p-Akt-1/2 and p-GSK-3β [7], [13], [28], [36]–[39]. Total cell lysates of BCBL-1 cells seeded with 100 µM nimesulide were collected at 2 h, 8 h, and 24 h and used to measure p-Akt-1/2 (serine 473; Ser473) and p-GSK-3β activation levels. Compared to 24 h treatment with DMSO, nimesulide down-regulated p-GSK-3β by about 60% and p-Akt 1/2 by about 25% (Fig. 7a and 7c). We further confirmed these findings by FACE assay for t-Akt-1/2, p-Akt-1/2, t-GSK-3β, and p-GSK-3β. At 8 h and 24 h post-treatment, nimesulide treatment significantly down-regulated p-Akt-1/2 by 66.9% and 54.1% (Fig. 7b) and p-GSK-3β by 73% and 60% (Fig. 7d), respectively, with no statistically significant effect on t-Akt (Fig. 7b) and t-GSK-3β (Fig. 7d).

### Nimesulide down-regulates survival and angiogenesis factors in BCBL-1 cells

PELs are known to be dependent on autocrine growth factors, especially B-cell survival factors IL-6 and IL-10 as well as angiogenesis factors such as VEGF-A and VEGF-C. We examined the gene expression of B cell proliferation inducing cytokine IL-10, VEGF-A, and VEGF-C in BCBL-1 cells at 24 h post-treatment with 100 µM nimesulide. At 24 h post-treatment with the drug, we observed a significant reduction of about 57% in VEGF-C (Fig. 7f) gene expression but not in VEGF-A (Fig. 7e) or IL-10 (Fig. 7g) genes.

### Nimesulide induced G1 arrest in BCBL-1 cells

**Figure 8:**
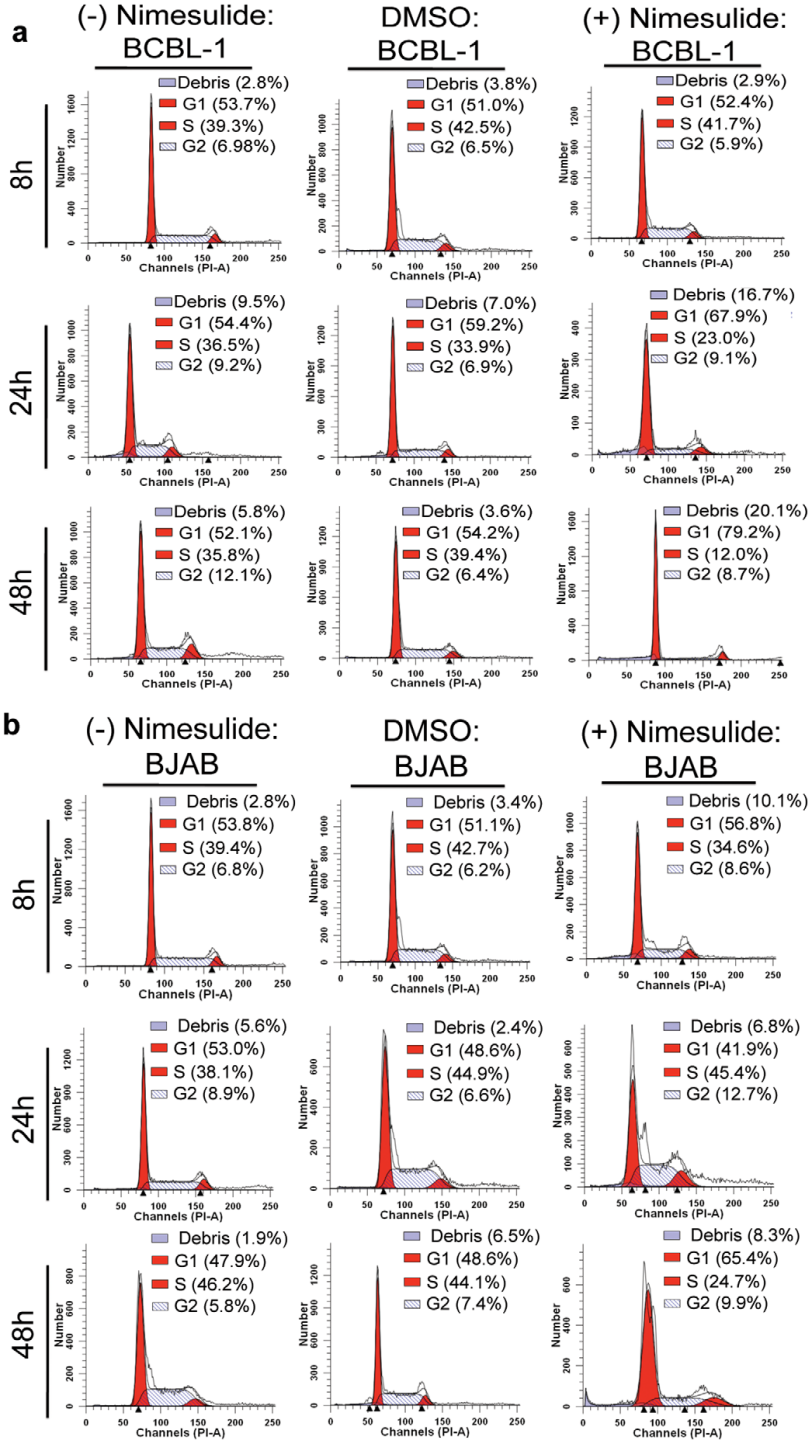
Effect of nimesulide on BCBL-1 cell cycle profile. (**a**) 5×10^5^ BCBL-1 and BJAB cells were seeded with 100 µM nimesulide or DMSO or left untreated without replenishment with fresh media. Samples were collected at 8 h, 24, and 48 h post-treatment to examine the cell cycle profile by propidium iodide (PI) staining. The cells were neither replenished with fresh media nor supplemented with the drugs. In each panel plotted, the horizontal and vertical axis corresponds to the relative DNA content and the number of cells, respectively. The percent of cells in G1, S, and G2 phases and the population of cellular debris for untreated (UN), DMSO treated, and nimesulide treated cells at the indicated time points was calculated by Modfit 3.2 software. The data represents three independent experiments.

BCBL-1 cells were seeded with 100 µM nimesulide and samples were collected at 8 h, 24 h, and 48 h post-treatment for examination by propidium iodide (PI) staining. At 8 h post-treatment, the population of cells in G1, S, G2 phases and population of debris were comparable between untreated (G1; 53.7%, S: 39.3%, G2; 6.98%; debris; 2.8%), DMSO (G1; 51.0%, S; 42.5%, G2; 6.5%; debris; 3.8%), and nimesulide (G1; 52.4%, S; 41.7%; G2; 5.9%; debris; 2.9%) treatments (Fig. 8a). At 24 h, nimesulide treatment increased the G1 population and debris of BCBL-1 cells to 67.9% and 16.7%, respectively compared to 8 h (Fig. 8a). A consequent decrease of the (G2+S) was seen for nimesulide treated cells (32.1% [23.0%+9.1%]) (Fig. 8a). At 48 h nimesulide treatment further increased the G1 population and debris of BCBL-1 cells to 79.2% and 20.1%, respectively with a consequent decrease of the (G2+S) population was seen for nimesulide treated cells (20.7% [12.0%+8.7%]) (Fig. 8a). However, the population of cells in G1, S, and G2 phases and the population of debris in DMSO treated and untreated BCBL-1 cells did not vary substantially between 8 h, 24 h, and 48 h. The population of cells in G1, S, G2 phases and population of debris were comparable between 8 h, 24 h, and 48 h post-treatment for untreated (24 h {G1; 54.4%, S: 36.5%, G2; 9.2%; debris; 9.5%} & 48 h {G1; 52.1%, S: 35.8%, G2; 12.1%; debris; 5.8%}) and DMSO (24 h {G1; 59.2%, S: 33.9%, G2; 6.9%; debris; 7.0%} & 48 h {G1; 54.2%, S: 39.4%, G2; 6.4%; debris; 3.6%}) treatment (Fig. 8a).

The BJAB cell cycle profile changed from 8 h to 24 h and 48 h post-nimesulide treatment from 56.8% to 41.9% and 65.4% for G1, 34.6% to 45.4% and 24.7% for S, and 8.6% to 12.7% and 9.9% for G2, and 10.1% to 6.8% and 8.3% for the population of debris, respectively (Fig. 8b). Simalarily, the population of cells in G1, S, G2 phases and population of debris were comparable between 8 h, 24 h, and 48 h post-treatments for untreated (8 h {G1; 53.8%, S: 39.4%, G2; 6.8%; debris; 2.8%}, 24 h {G1; 53.0%, S: 38.1%, G2; 8.9%; debris; 5.6%} & 48 h {G1; 47.9%, S: 46.2%, G2; 5.8%; debris; 1.9%}) and DMSO (8 h {G1; 51.1%, S: 42.7%, G2; 6.2%; debris; 3.4%}, 24 h {G1; 48.6%, S: 44.9%, G2; 6.6%; debris; 2.4%} & 48 h {G1; 48.6%, S: 44.1%, G2; 7.4%; debris; 6.5%}) treatment (Fig. 8b). Overall, nimesulide treatment substantially increased the population of G1 and debris on BCBL-1 with a consequent decrease in the proliferative population of S and G2 (Fig. 8b).

### Nimesulide induces apoptosis in BCBL-1 cells

**Figure 9:**
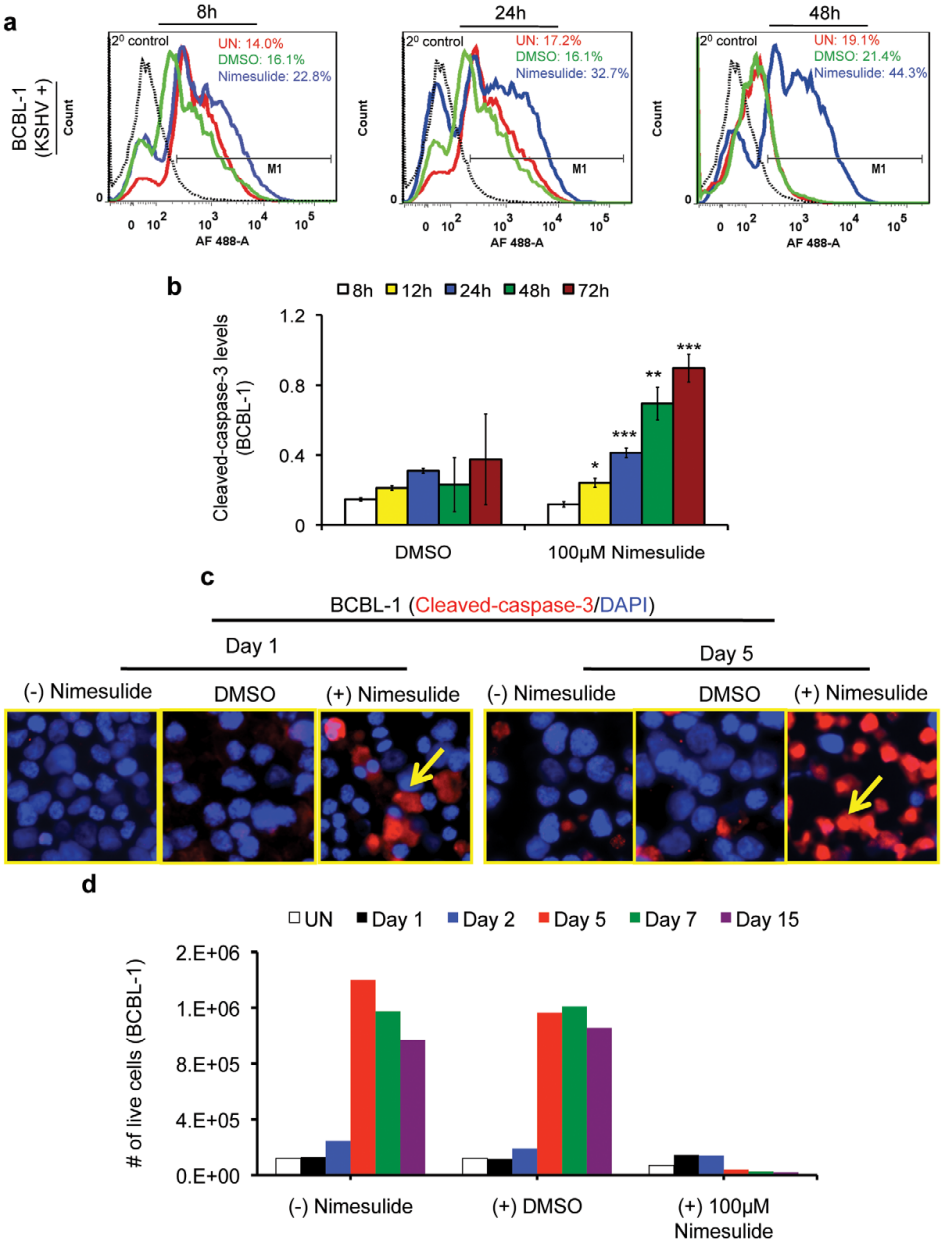
Effect of nimesulide on the apoptotic marker cleaved-caspase-3 in BCBL-1 and BJAB cells. (**a**) 5×10^5^ BJAB and BCBL-1 cells were seeded with 100 µM nimesulide or DMSO or left untreated,. Samples were collected at 8 h, 24 h, and 48 h to examine the levels of the apoptotic marker cleaved-caspase 3 by FACS. The cells were neither replenished with fresh media nor supplemented with the nimesulide. Expression of cleaved caspase-3 is shown as compared to secondary alone control, which is shown in black (dotted) histogram. (**b**) The effect of nimesulide on cleaved-caspase-3 levels was measured by a more sensitive colorimetry based apoptosis assay as well at identical conditions as Fig. 9a at 8 h, 12 h, 24 h, and 72 h post-treatment with nimesulide. (**c**) In parallel experiments, BCBL-1 cells were collected at 24 h and day 5 after drug supplementation at days 3 and 4 to determine the levels of cleaved-caspase 3 by immunofluorescence for nimesulide and DMSO treatments, and for cells left untreated. (**d**) 1×10^5^ BCBL-1 cells were seeded with 100 µM nimesulide and samples were collected at days 1, 2, 5, 7, and 15 to measure the number of live cells. 100 µM nimesulide was supplemented at days 3 and 4. The drug was removed by washing with PBS at day 7 and cells were replenished with fresh media. (**a–d**) The data represents four independent experiments. (*) p<0.001, (**) p<0.00001), (***) p<0.000001).

BCBL-1 and BJAB cells seeded with 100 µM nimesulide collected at 8 h, 24 h, and 48 h were analyzed by FACS to measure the levels of cleaved-caspase 3, an apoptotic marker. At 8 h, 14% of untreated, 16.1% of DMSO, and 22.8% of nimesulide treated BCBL-1 cells were positive for cleaved-caspase 3 (Fig. 9a). At 24 h, cleaved-caspase-3 levels increased modestly to 32.7% for nimesulide treated compared to 17.2% and 16.1% for untreated and DMSO treated cells, respectively (Fig. 9a). In contrast, at 48 h, BCBL-1 cells positive for cleaved-caspase-3 dramatically increased to 44.3% for nimesulide compared to 19.1% and 21.4% for untreated and DMSO cells, respectively (Fig. 9a). We further verified the pro-apoptotic effects of nimesulide on BCBL-1 by demonstrating the increase in cleaved-caspase-3 levels at 8 h, 12 h, 24 h, 48 h, and 72 h post-treatment with nimesulide using a colorimetry based assay (Fig. 9b). Immunofluorescence observations also confirm the induction of apoptosis by nimesulide supplementation at day 5 (Fig. 9c). Next, we examined the effect of nimesulide on the population of viable BCBL-1 cells.BCBL-1 cells seeded with 100 µM nimesulide were collected at days 1, 2, 5, 7, and 15 to measure the number of live cells using the traditional trypan blue exclusion assay (Fig. 9d). Cells were supplemented with 100 µM nimesulide at days 3 and 4. The drug was removed at day 7 and the cells were replenished with fresh media. Compared to the increase in the number of live cells for DMSO treated and untreated cells, nimesulide substantially decreased the population of live cells after drug supplementation.

### The COX-2/PGE2 pathway is up-regulated in BCBL-1 compared to BJAB cells and nimesulide down-regulates PEL specific genes

**Figure 10:**
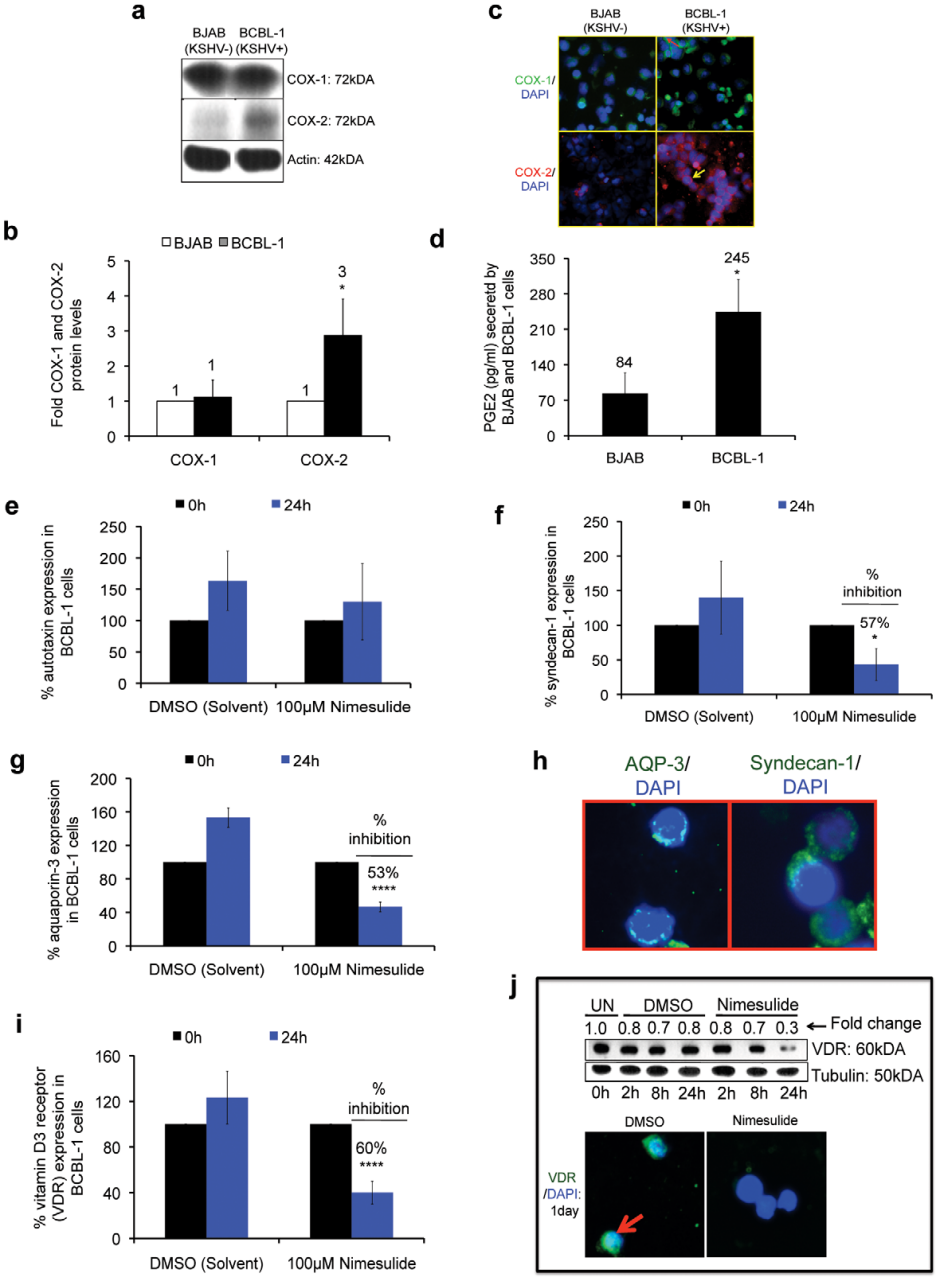
COX-2/PGE2 status in BCBL-1 cells and effect of nimesulide on PEL specific genes. (**a–b**) Total lysates from 5×10^5^ BCBL-1 and BJAB cells were immunoblotted (**a**) for COX-1 and COX-2. (**b**) Protein band intensity was measured by densitometry and normalized with respect to actin to quantitate the relative differences and the fold difference was calculated with respect to BJAB cells. (**c–d**) In parallel experiments, COX-1/COX-2 protein levels and secreted PGE2 in the supernatant of BCBL-1 and BJAB cells were examined by immunofluorescence (**c**) and ELISA (**d**), respectively. The fold change and statistics (t-test) were calculated with respect to BJAB cells. (**b, d**) Each experiment was done in tripicate, and each point represents the average ± S.D. from three independent experiments. (*) p<0.05. (**e, f, h, i**) 5×10^5^ BCBL-1 cells were seeded with 100 µM nimesulide or DMSO or left untreated without replenishment with fresh media. RNA was isolated at 24 h post-treatment with the drug to examine the gene expression levels of aquaporin-3 (**e**), syndecan-1 (**f**), autotoxin (**h**), and VDR (**i**) by real-time RT-PCR. The cells were neither replenished with fresh media nor supplemented with the drugs. The fold change and corresponding statistics (t-test) were calculated with respect to untreated cells for each treatment. Each experiment was done in tripicates, and each point represents the average ± s.d. from three independent experiments. (*) p<0.05, (****) p<0.00005. (**g**) The cellular distribution of aquaporin-3 and syndecan-1 in BCBL-1 cells was examined by immunofluorescence. (**j**) 5×10^5^ BCBL-1 cells were seeded with 100 µM nimesulide or DMSO and total lysates were isolated at 2 h, 8 h, 24 h post-treatment to determine the protein levels of VDR by Western blotting (top panel). In parallel experiments, the effect of 100 µM nimesulide or DMSO on VDR cellular distribution was examined by immunofluorescence 24 h post-treatment with the drug (bottom panel).

We next compared the status of the COX-2/PGE2 pathway in BJAB and BCBL-1 cells. Western blot analysis demonstrated that compared to BJAB cells, COX-2 (Fig. 10a) but not COX-1 (Fig. 10a) was significantly up-regulated in BCBL-1 cells by 3-fold (Fig. 10b). Immunofluorescence analysis also exhibited a stronger signal for COX-2 but not COX-1 in BCBL-1 cells relative to BJAB cells (Fig. 10c). Similarly, a significant quantity of PGE2 was secreted from BCBL cells (245 pg/ml) compared to BJAB cells (84 pg/ml) (Fig. 10d).

Previous studies have shown that unlike other NHLs, PEL is characterized by the overexpression of aquaporin-3, syndecan-1, autotaxin, and aquaporin-3 [40], [41]. To examine whether the anti-KSHV specific properties of nimesulide are due to the effect of COX-2 blockade on these PEL specific genes, we next analyzed the gene expression profile of autotaxin (Fig. 10e), syndecan-1 (Fig. 10f), aquaporin-3 (Fig. 10g), and VDR (Fig. 10i) with nimesulide treatment. At 24 h post-treatment with the drug, we observed a significant reduction of about 53%, 57%, and 60% in aquaporin-3 (Fig. 10g), syndecan-1 (Fig. 10f), and VDR (Fig. 10i) respectively but no effect on autotaxin (Fig. 10e). The surface level staining of aquaporin-3 and syndecan-1 was also confirmed by immunofluorescence (Fig. 10h). In parallel experiments, we also demonstrated that nimesulide down-regulated VDR protein levels by about 70% (Fig. 10j; top panel) and its nuclear translocation (Fig. 10j; bottom panel) at 1-day post-treatment.

## Discussion

PEL is a rare NHL commonly arising in the pleural, pericardial, or peritoneal spaces and displays evidence of KSHV infection. PEL is an aggressive NHL and even with combination chemotherapy prognosis remains quite poor with median survival times of only about 6 months with very few long-term survivors. However, with better understanding of the unique oncogenesis of PEL, it is hoped that rational and specific targets for therapy can become the basis for greater therapeutic success. Therefore, we embarked on our study with the goal of understanding the role of pro-inflammatory angiogenic stress response gene COX-2 in PEL pathogenesis, if any, and finding an NSAID with anti-KSHV, anti-inflammatory, and anti-cancer effects to treat PEL. We calibrated our investigation with such specifics because of the cumulative interdependent vitality of the expression of KSHV latency genes, the pro-inflammatory environment, and the manipulation of canonical anti-cancer host defense machinery such as p53 and p21 in the metamorphosis of PEL neoplasia [1], [2]. We chose to explore the potential of COX-2 as an ideal chemotherapeutic target for achieving such a goal due to the well established tumorigenic potential of COX-2, the availability of well characterized COX-2 inhibitors with known anti-cancer effects and the correlation between COX-2 expression and poor NHL prognosis [24], [26], [28]–[29]. Treatment with 25–100 µM celecoxib (COX-2 inhibitor) for 48 h had been reported to inhibit proliferation of a few NHL cell lines [42] but has never been studied in KSHV+ PEL cell lines especially with respect to their effect on viral life cycle/latency. The novelty and importance of our study is the discovery of the link between the pivotal pro-inflammatory protein COX-2 and PEL latency and therefore the identification of COX-2 as a chemotherapeutic target and the prescription drug nimesulide in treating PEL.

Overall, nimesulide had anti-proliferative effects on all the cell lines tested. This was not surprising since all the cell lines tested are NHL cell lines with potential proliferative advantages provided by COX-2 mediated inflammatory mechanisms. Therefore, an important question that was examined to confirm the role of COX-2 in KSHV mediated PEL pathogenesis was whether the KSHV specific anti-latency/survival and non-specific anti-survival effects of nimesulide mediated COX-2 blockade can be delineated, if any. Our data demonstrating the statistically significant difference in the proliferation index of KSHV-BJAB and BJAB suggests that the presence of KSHV infection made BJAB more vulnerable to COX-2 blockade mediated anti-proliferative mechanisms, since KSHV-BJAB is a cell line created by the introduction of a recombinant KSHV virus to BJAB cells, which was resilient to nimesulide mediated apoptosis even at 100 µM nimesulide. This observation is further augmented by the significant difference in the proliferation index of KSHV+/EBV+ (JSC-1) and KSHV+/EBV- (BCBL-1, BC-3, KSHV-BJAB) cell lines with KSHV-/EBV-Burkitt's lymphoma cells (BJAB, Ramos, Akata/EBV-) 1 day post-treatment with 50 µM nimesulide. Despite encouraging observations, final conclusions have to be drawn with caution as there are some caveats to interpret data from the artificial, not “naturally infected” cell line such as KSHV-BJAB. KSHV-BJAB is a BJAB-derived cell line, which was created by nucleofection of KSHV-negative BJAB cells with the KSHV bacterial artificial chromosome containing a hygromycin antibiotic resistance marker and the GFP expression cassette. Since this cell line is maintained by drug selection unlike the parent cell line BJAB, can make their comparisons questionable. Ideally, both cell lines should have been cultured under selection and then treated with nimesulide or solvent control. Although, statistically significant differences between the proliferation rate of KSHV+ and KSHV-/EBV- cells were observed, the cells we used were all from NHL cell lines and are therefore not ideal complementary control cell lines. Therefore, we further addressed the question of whether the anti-proliferative effect of nimesulide is due to the general cytotoxic effect of the drug on transformed cells or due to a KSHV specific effect by comparing the effect of nimesulide on the proliferation of TIVE-LTC (KSHV+), telomerised TIVE (KSHV-), primary endothelial (HMVEC-d) cells. Primary endothelial (HMVEC-d) and TIVE cells were resistant to the anti-proliferative effects of nimesulide (50 µM and 100 µM) compared to KSHV infected endothelial cells (TIVE-LTC). Thus, our data (Figs. 1, 2, 3, 4a-4d and table 1) suggests that nimesulide mediated anti-proliferative effects on PEL are due to the additive effects of blocking KSHV latency and COX-2 mediated cell survival mechanisms.

90% of PEL cell lines are co-infected with KSHV and EBV and therefore it was imperative to test whether the anti-proliferative effects of nimesulide on KSHV+/EBV- cell lines is replicable on PEL cell lines co-infected with KSHV and EBV. The significant difference in the proliferation index of JSC-1 and KSHV-/EBV+ (LCL) and KSHV-/EBV- cells, along with the absence of a similar effect between LCL and KSHV-/EBV- cells, strongly suggests that the anti-proliferative effects of nimesulide is due to a specific anti-KSHV effect but not an anti-EBV effect (Figs. 1, 2, 3, and table 1). Although, our data does not demonstrate a specific anti-EBV effect, further work needs to be done with lower concentrations of nimesulide to determine whether nimesulide also holds specific anti-EBV properties. Although, previous reports have demonstrated the anti-cancer effects of other COX-2 inhibitors, such as etodolac in EBV+ Burkitt's lymphoma cell lines [43]–[44], ours is the first work to demonstrate the chemotherapeutic potential of the prescription COX-2 inhibitor nimesulide in treating KSHV associated NHLs and in down-regulating the genes of the etiologic agent.

The chemotherapeutic potency of any anti-cancer drug relies on its selective toxicity to cancer cells. The elevated levels of COX-2 in BCBL-1 cells might explain the potency of selective anti-growth effects of nimesulide on these cells in terms of proliferation, apoptosis induction and cell viability. In addition, our observation of elevated COX-2 levels in BCBL-1 cells might correlate with the overall aggressiveness of PEL, since COX-2 expression has often been associated with the poor response to chemotherapy, dismal survival outcomes, and recurrence of NHLs [24]. Our study further explores the functional consequences of COX-2 up-regulation in BCBL-1 cells in order to understand the mechanistic themes underlying the selective toxicity of COX-2 inhibitor nimesulide to BCBL-1 cells. The induction of proliferation arrest, alteration in cell cycle profile, and cell death by nimesulide could be related to the down-regulation of KSHV latency proteins LANA-1 and vFLIP as well as to the down-regulating effect of blocking COX-2 on cell survival proteins such as VEGF-C, IL-10, p-GSK-3β, and p-Akt independently of viral proteins (Figs. 5–7) [3], [4], [13], [15]–[20], [36], [38], [45]–[48].

The up-regulation of p21, release of p53 interaction with LANA-1, and consequent changes in cell cycle profile and cell cycle proteins along with the increase in cleaved-caspase-3 levels by nimesulide treatment indicates an initiation of G1 arrest followed by the induction of apoptosis (Figs. 6 and 8). Thus, the potency of nimesulide lies in its capacity to provide a fully transformed B cell such as BCBL-1 with a chance for the inherent anti-cancer host mechanisms to self-initiate cell cycle arrest and programmed cell death by blocking the viral and host mechanisms utilized by the virus to overcome such mechanisms, which is strongly suggested by the reduction in the colony forming capacity of BCBL-1 cells by the drug treatment (Fig. 4e, 4f).

PEL is believed to have a unique NHL gene expression profile with the overexpression of plasma cell marker syndecan-1, VDR, and aquaporin-3 [40], [41]. Recent reports have shed light on the role of the transmembrane proteoglycan syndecan-1 in different aspects of oncogenesis such as cell migration through Rac-1/PKCα signaling [49]. VDR is the natural receptor for 1α,25-dihydroxyvitamin D3 and its activation is associated with chromatin remodeling and is also proposed to increase the risk of esophageal squamous, prostate, and pancreatic cancers [50]. Aquaporin-3 is a channel protein involved with the transportation of water and glycerol, ATP generation, and is proposed to be important in tumorigenesis by promoting cell migration and energy nourishment for proliferation [51]. Thus, our data (Fig. 10) demonstrating the down-regulation of syndecan-1, aquaporin-3, and VDR by nimesulide provides an additional mechanistic framework to understand the specific anti-PEL specific effects of nimesulide and also exposes novel pathways such as proteoglycan mediated signaling, chromatin remodeling, and ATP metabolism by which COX-2 might be promoting oncogenesis in other cancer systems.

**Figure 11:**
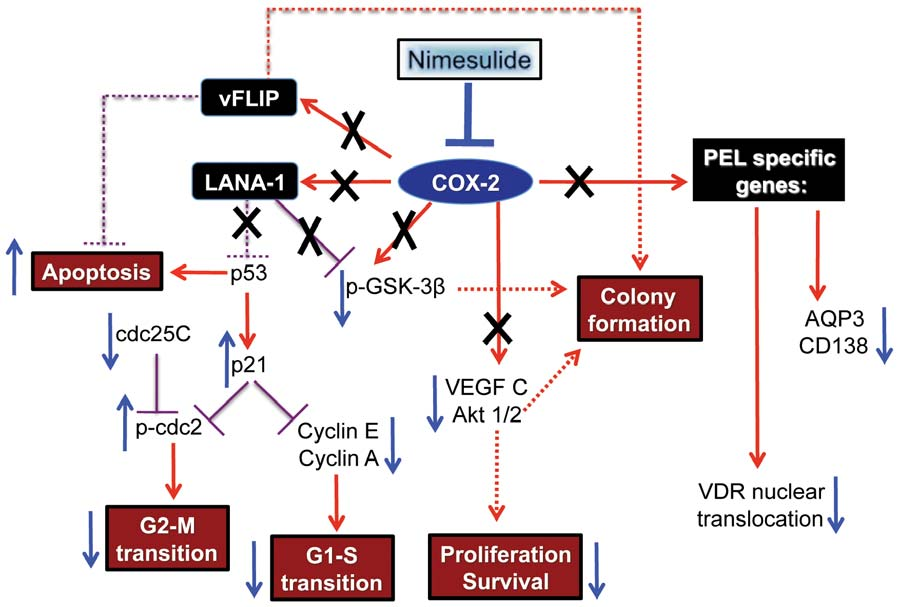
Schematic model representing the mechanistic effect of the COX-2 inhibitor nimesulide on PEL pathogenesis. COX-2 blockade by nimesulide inhibits latency genes LANA-1 and vFLIP. The pro-survival mechanisms that COX-2 provides PEL cells through viral gene independent and dependent pathways, such as proliferation, cell cycle regulation, apoptosis blockade, angiogenesis, and metastasis are blocked by nimesulide through the alteration of cell survival and PEL specific genes as indicated.

Like other aggressive cancers, PEL is associated with cell proliferation, immune evasion, and anti-apoptotic mechanisms resulting from the deregulation of multiple proteins. However, unlike many cancers, PEL oncogenesis also depends on KSHV latency that appears to be maintained by various pro-inflammatory proteins such as COX-2 [25], [27]–[28]. PEL pathogenesis is hypothesized to be the eventuality of the persistence of mutually inclusive interdependent interactions between KSHV latency, inflammation, immune suppression, and various tumorigenic mechanisms. Thus, the specificity, the potency, and the sustainability of the ‘knock-out’ punch of nimesulide on PEL cells could be attributed to additive anti-viral effects by the blockade of KSHV latency genes LANA-1/vFLIP, anti-inflammatory/anti-survival properties by the down-regulation of VEGF-C, Akt1/2, and GSK-3β, anti-PEL specific properties by down-regulating syndecan-1, VDR, and aquaporin-3 as well as anti-cancer properties by the activation of G1 arrest and apoptosis (Fig. 11). While it is true that the dose of nimesulide we used (100 µM) appears higher than the FDA approved dose for the treatment of arthritis or familial adenomatous polypopsis, the maximum tolerated dose for the cancer treatment in humans has not been established yet. Studies in murine breast cancer treatment models have established a dose of 20–25 mg/kg of COX-2 inhibitor (celecoxib) as the maximum tolerated dose which results in blood levels in the 400–700 µM range, eight to ten times higher than the in vitro doses used in our study [52]. In addition, the reported *in vitro* IC50 of nimesulide for most cancer cell lines has been reported to be more than 150 µM–175 µM. We chose 100 µM because tukey's posthoc comparison analysis of the mean proliferative indexes at day 1 between the different concentrations of nimesulide demonstrated that 100 µM had a more significant decrease in the proliferative capacity of BCBL-1 cells compared to 50 µM with no effect on BJAB cells.

Overall, our study is providing a novel framework to understand PEL pathogenesis using the well-known COX-2 inhibitor nimesulide and is thus paving the way for a novel arena of chemotherapeutic drugs to develop a potent treatment for PEL. However, the anti-proliferative effects of other NSAIDs, such as indomethacin and diclofenac in addition to nimesulide, indicates that a combination of NSAIDs at much lower concentrations might be equally or more selectively potent against PEL cells. Further work needs to be done using animal models to evaluate such an idea. Currently, NHLs are the fifth most common cancer in the US and account for 5% of all cancers with an annual incidence increasing by 1–2% [5], [53]. Therefore, we also predict that our study investigating the role and chemotherapeutic potential of blocking COX-2 using nimesulide would be an applicable model for other NHLs similar to PEL, including Burkitt's lymphoma and diffuse large B cell lymphoma [1].

## Supporting Information

Figure S1 **Effect of nimesulide on KSHV infected endothelial cells.** (**a-c**) TIVE (a), TIVE-LTC (b), and HMVEC-d (c) cells were serum starved for 48 h and treated with the indicated concentrations of nimesulide and cell proliferation was measured by MTT assay at day 1 (1d; a), day 3 (3d; c), and day 4 (4d; d). The cells were neither replenished with fresh media nor supplemented with the drugs. Each experiment was done in tripicates, and each point represents the average ± s.d. from three independent experiments. One-way ANOVA with Tukey's posthoc comparison analysis (p<0.05) was used to determine whether the drug treatment induced a statistically significant difference in the proliferative indexes at 1d, 3d, and 5d compared to untreated cells of the respective cell lines.
(TIF)
