# Supplementary figures and images for: Targeting KSHV/HHV-8 Latency with COX-2 Selective Inhibitor Nimesulide: A Potential Chemotherapeutic Modality for Primary Effusion Lymphoma
Source: PLoS One. 2011 Sep 30;6(9):e24379. doi: 10.1371/journal.pone.0024379 (PMC3184084; doi:10.1371/journal.pone.0024379)

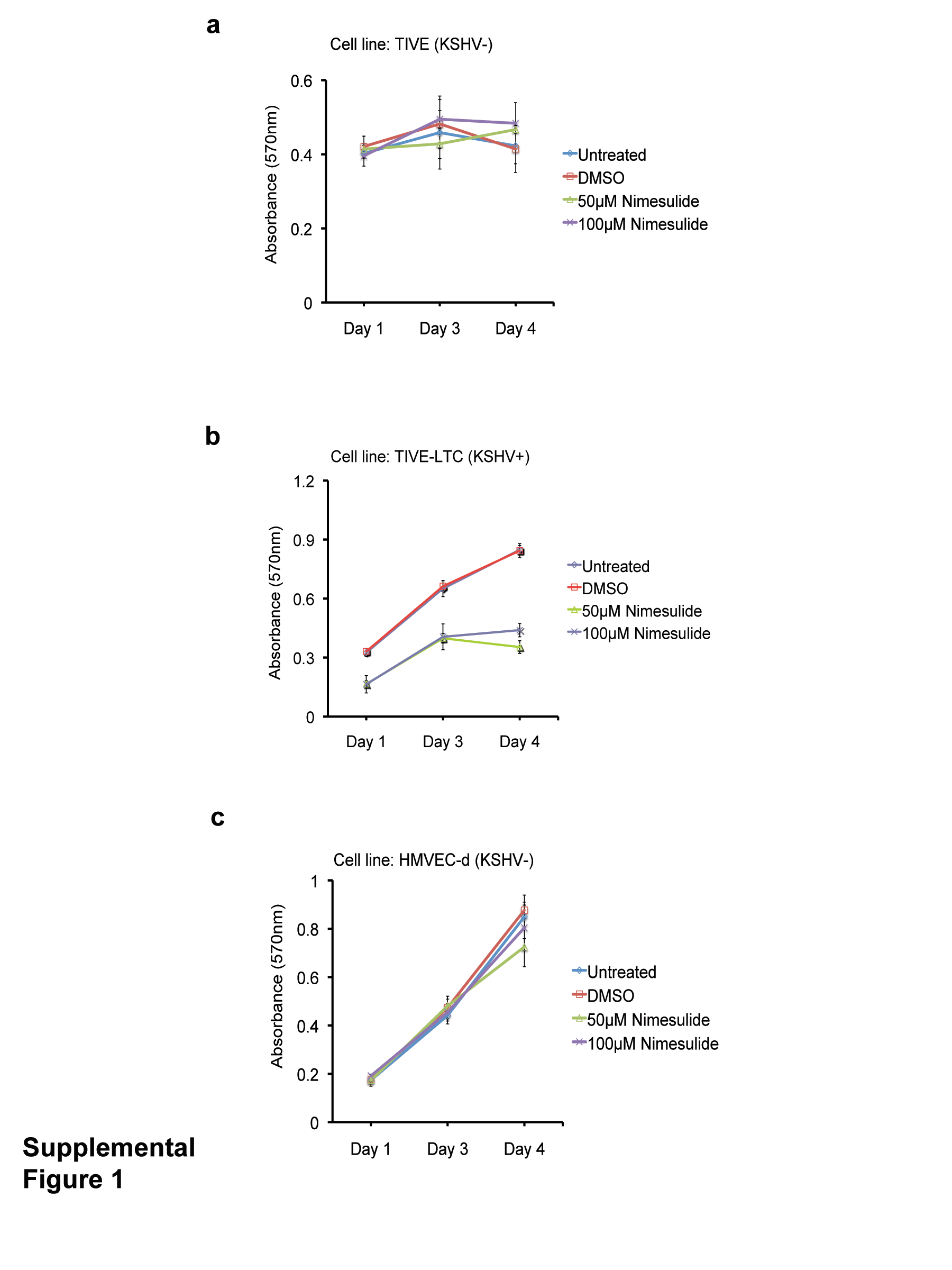

Supplement: Figure S1 — Effect of nimesulide on KSHV infected endothelial cells. (a-c) TIVE (a), TIVE-LTC (b), and HMVEC-d (c) cells were serum starved for 48 h and treated with the indicated concentrations of nimesulide and cell proliferation was measured by MTT assay at day 1 (1d; a), day 3 (3d; c), and day 4 (4d; d). The cells were neither replenished with fresh media nor supplemented with the drugs. Each experiment was done in tripicates, and each point represents the average ± s.d. from three independent experiments. One-way ANOVA with Tukey's posthoc comparison analysis (p<0.05) was used to determine whether the drug treatment induced a statistically significant difference in the proliferative indexes at 1d, 3d, and 5d compared to untreated cells of the respective cell lines. (TIF) [file pone.0024379.s001.tif]
